# Effects of effort-reward imbalance on emergency nurses’ health: a mediating and moderating role of emotional exhaustion and work-family conflict

**DOI:** 10.3389/fpubh.2025.1580501

**Published:** 2025-05-26

**Authors:** Yuanyuan Tan, Jing Zhou, Hao Zhang, Lin Lan, Xiaoli Chen, Xiaomin Yu, Luying Zhong, Ling Zhu, Yongli Gao

**Affiliations:** ^1^Department of Health Management Center, Sichuan Cancer Hospital & Institute, Sichuan Cancer Center, University of Electronic Science and Technology of China, Chengdu, China; ^2^Department of Emergency Medicine, West China Hospital, Sichuan University/West China School of Nursing, Sichuan University, Chengdu, China; ^3^Institute of Disaster Medicine, Sichuan University, Chengdu, China; ^4^Nursing Key Laboratory of Sichuan Province, Chengdu, China

**Keywords:** effort-reward imbalance, somatic symptoms, sleep disorders, emergency department nurses, mediation model

## Abstract

**Background:**

Effort-reward imbalance (ERI) is a prevalent issue in the healthcare sector, particularly in the high-intensity, high-risk, and high-stress environment of the emergency department. This results in emergency department nurses bearing heavier workloads, responsibilities, and time commitments compared to their counterparts in other departments. ERI poses significant risks to their physical, psychological, and sleep quality. Therefore, it is essential to explore the mechanisms through which ERI influences the health of emergency department nurses.

**Objective:**

The aim of this investigation is to analyze if work–family conflict moderates the mediating influence emotional exhaustion has on the association between ERI and somatic symptoms and sleep disorders.

**Design:**

A cross-sectional study.

**Settings:**

The emergency nurses (*N* = 1,540) were included from 30 tertiary hospitals in 20 provinces or autonomous regions (Northeast, North, East, Central, South, Southwest, and Northwest China) of mainland China between December 26, 2023, and January 18, 2024.

**Methods:**

Participants were recruited using stratified cluster sampling, obtaining data through web-based questionnaires. The study investigated the mediating and moderating effects using the *PROCESS* macro for SPSS. The mediation effect is tested by the bias correction *Bootstrap* sample size was set to 5,000.

**Result:**

Considering emotional exhaustion as a mediating variable, the direct predictive influence of ERI on somatic symptoms and sleep disorders continues to be statistically significant (*β* = 0.271, 0.137, *p* < 0.01). Compared to the high-level work–family conflict group, the positive moderating effect of low-level work–family conflict on the relationship between ERI and emotional exhaustion was more pronounced (*simple slope* = 0.479, 0.757, *p* < 0.01). The moderated mediation effects of emotional exhaustion on somatic symptoms and sleep disorders are −0.063 (95%CI: −0.077 ~ −0.050) and −0.044 (95%CI: −0.056 ~ −0.033) respectively.

**Conclusion:**

The study findings indicate that ERI was correlated with heightened emotional exhaustion, somatic symptoms, and sleep disorders among emergency department nurses. As a result, interventions should be implemented to improve ERI, alleviate emotional exhaustion among nurses, monitor work–family conflict levels, and mitigate the effects of these factors on nurses’ overall well-being.

## 1 Introduction

Effort-reward imbalance (ERI) refers to an individual’s perception of the disparity between the efforts expended and the rewards received in the workplace (1). The balance between effort and reward will prompt employees to have a correct understanding of their work investment and increase their occupational well-being. It also holds great significance for enhancing organizational fairness and organizational production efficiency (63). Emergency department nurses are crucial in the emergency medical service system and are facing significant challenges (2–4). They frequently lack sufficient job compensation, respect, and professional recognition. Such circumstances can give rise to a high-effort, low-return scenario, which not only undermines their job satisfaction but also poses potential risks to the quality of emergency medical services (5–7). Studies from mainland China (8, 9) have shown a 59.66% incidence of ERI among emergency nurses, higher than nurses in general departments. Bardhan et al. found that the risk of ERI among emergency nurses in the United States was over 90% (10). Weyers et al. identified a link between nurses receiving both high effort-low return and experiencing cardiovascular, gastrointestinal, and musculoskeletal health issues (11). Deng et al.’s study further validated that elevated levels of ERI can significantly influence the sleep quality of community nurses (12). Yu et al. demonstrated that ERI is linked to the potential suboptimal health status for nurses, leading to the manifestation of symptoms across various domains, including fatigue, cardiovascular issues, digestive problems, compromised immune function, and mental well-being (13). These conclusions emphasize the urgent need to further explore the impact pathways of ERI on the health outcomes of emergency department nurses.

Research has found that the fundamental essence of emotional exhaustion serves as the most effective definition of burnout (14). The discrepancy between effort and reward at work leads to emotional distress, with ERI serving as a significant predictor of emotional exhaustion (15, 16). Emotional exhaustion, a key component of burnout, arises when an individual’s psychological and emotional resources are depleted due to workplace stressors (14, 17). Notably, the prevalence of emotional exhaustion among emergency department nurses was found to be 40.5%, significantly higher than in other departments (18, 19). Studies indicates a strong connection between emotional exhaustion and factors such as fatigue linked to stress, depression associated with work, psychosomatic issues, and anxiety (20). Alvarado et al. revealed that nurses’ effort directly correlates with emotional exhaustion, while reward indirectly affects emotional exhaustion through work experience (21). A systematic review indicates that reducing emotional exhaustion can enhance the health of healthcare workers (22).

Work–family conflict (WFC) is another significant potential moderating factor adversely affecting nurse health outcomes. Emergency department nurses not only contend with heavy workloads, prolonged stress, work interruptions, and responsibilities, but also require strong motivation and emotional involvement, which presents significant challenges in balancing family and work commitments (23). Kinman et al. identified that the effort, reward, ERI, and overcommitment influence employees’ experience of WFC (24). Ghorpade highlighted that role conflict and role ambiguity are key contributors to emotional exhaustion (25). Innstrand et al. discovered a delayed positive relationship between WFC and emotional exhaustion in a longitudinal study involving various occupational groups like teachers, doctors, and nurses (26). The study revealed that WFC depletes individuals’ physical and mental resources, exacerbating emotional exhaustion (27). WFC is fundamental aspect of life for individuals in any profession. Research by Wang et al. indicates that this conflict can result in significant emotional exhaustion among nurses (28). However, there is limited research on how WFC moderates the relationship between ERI and emotional exhaustion among emergency department nurses.

Siegrist et al. developed the ERI model to examine the impact of the non-reciprocal relationship between efforts and rewards on individual health and stress outcomes (29). The model posits that individuals invest time and energy at work with the expectation of receiving rewards such as money, respect, and status from the organization. This study aimed to fill this gap by exploring the interaction between ERI and occupational health among emergency nurses, with a particular focused on the mediating role of emotional exhaustion and the moderating effect of WFC. We hypothesized that: (I) ERI, emotional exhaustion, WFC, and occupational health are interrelated; (II) emotional exhaustion mediation affects the relationship between ERI and nurse health; and (III) Work-family balance can moderate the impact of ERI on occupational stress and nurse health.

## 2 Methods

### 2.1 Study design and participants

A cross-sectional survey was conducted from 30 tertiary hospitals in 20 provinces or autonomous regions of mainland China between December 26, 2023, and January 18, 2024. Participants were recruited using stratified cluster sampling. The stratification was based on the geographical regions of China (Northeast, North, East, Central, South, Southwest, and Northwest China). Two to three hospitals were selected in each city, which mainly include provincial capital cities, prefecture-level cities and municipalities. Inclusion criteria required participants to hold a nursing license, be actively working in the emergency department for over a year, and provide informed consent to participate voluntarily. Exclusion criteria encompassed emergency nurses undergoing standardized or further training, as well as those who were not on duty during the survey.

The research team has previously published the path mechanisms of the influences of different factors on the health of emergency nurses. Stratified cluster sampling was used to select public hospitals across China. Based on a test level of 0.05 and a test efficiency of 80%, the required sample size for the preliminary pre survey calculation was 1,330 cases (30, 31). A total of 1,555 emergency department nurses participated the survey. When the researchers discovered duplicate IP addresses, years of working inconsistencies in claimed age and all options yielded identical answers, the authors excluded such investigation data from the sample. Excluding 15 incomplete responses to the questionnaire, leaving an effective sample at 1540 participants.

### 2.2 Measuring tools

A self-designed basic information questionnaire was utilized to gather data on the demographic and sociological information (gender, marital status, number of children, educational level), work-related characteristics (professional title, years of nursing experience, night shift, working hours per week, number of night shift), and lifestyles (smoking status, drinking status) of emergency department nurses.

The ERI of emergency department nurses was evaluated using the ERI questionnaire developed by Siegrist, based on the ERI model (16). The Chinese version of the ERI scale was translated revised by Chinese scholar Junming Dai, demonstrating good reliability and validity (32). The results of the scale reliability analysis of the data in this study are as follows. The Cronbach’s *α* coefficient of the scale is 0.957, three dimensions are 0.894, 0.952, and 0.922, respectively. The scale consists of 6 items for effort, 11 items for reward, each rated on a Likert scale from 1 to 5. The ERI index is calculated by dividing the effort score by the product of the reward score and 6/11. Specifically, an ERI index exceeding 1.0 signifies an imbalanced condition characterized by high effort and low return.

The Maslach Burnout Inventory-General Survey (MBI-GS), developed by Maslach et al. (17), was utilized to evaluate the current level of emotional exhaustion among emergency department nurses. The Chinese version of the scale was translated and adapted by Chinese scholars Huang et al. (33). The emotional exhaustion dimension comprises 5 items, each rated on a 7-point Likert scale from 0 to 6, with higher scores indicating greater emotional exhaustion. The results of the scale reliability analysis of the data in this study are as follows. The Cronbach’s *α* coefficient of the scale is 0.904, and Cronbach’s α coefficient for emotional exhaustion dimension of 0.964.

The Self-administered Sleep Questionnaire (SSQ), developed by Japanese scholar Nakata, was utilized to assess sleep disorders among nurses in the emergency department (34). The questionnaire evaluated three categories of sleep symptoms—time to fall asleep, persistent sleep, and early morning awakening—through three items. Each item was scored on a scale from 1 to 5, with the total score being the sum of these scores. A higher total score indicated a more severe degree of sleep disorders. The results of the Cronbach’s *α* coefficient analysis of the data in this study is 0.809.

The health of emergency department nurses was evaluated using the Chinese version of the Somatization Symptom Self-Rating Scale-China (SSS-CN) developed by Jiang et al. (35). This scale comprises 20 items, with 10 items focusing on physical disorder and the other 10 items covering the psychological disorder aspects such as anxiety, depression, and anxiety-depression, enabling a thorough evaluation of the patient’s psychological, behavioral, and somatization symptoms. Each item was rated on a scale of 1 to 4 using the Likert 4-point scale. The results of the scale reliability analysis of the data in this study are as follows. The Cronbach’s *α* coefficient of the scale is 0.974, two dimensions are 0.951, and 0.954, respectively.

The Work-Family Behavioral Role Conflict Scale (WFBRC-S), developed by Clark et al., was utilized in this study. The Chinese version of the scale was translated and revised by Zhang, a respected Chinese scholar, demonstrating strong reliability and validity (36, 37). Consisting of 19 items, each rated on a 5-point Likert scale from 1 to 5, higher scores on the scale indicate greater levels of role conflicts. The results of the scale reliability analysis of the data in this study are as follows. The Cronbach’s coefficient for the Work-Family Behavioral Role Conflict Scale was calculated to be 0.969.

### 2.3 Data collection

Data collection was conducted through an online questionnaire distributed via the Wenjuanxing platform.^1^ Prior to the official release, a pilot survey was carried out in two tertiary hospitals in Chengdu to address any technical issues. The Emergency Nursing Specialty Committee of the Chinese Nursing Association supported the study, with nurse administrators in the emergency departments contacted to explain the study’s purpose and obtain consent. Eligible emergency nurses received the questionnaire link via WeChat, ensuring voluntary participation and anonymity. To prevent duplicate submissions, each IP address was limited to one response. Questionnaires with identical answers or inconsistent respondent information were excluded.

### 2.4 Statistical analysis

Statistical analysis was conducted using SPSS 25.0 software and *PROCESS* macro. Continuous data that conforms to a normal distribution was described as ( $\bar{x}\pm s$ ) and categorical data as *n* (%). Pearson correlation analysis was employed to investigate the initial relationships between dimensions and factors within the model. The mediation and moderated mediation model were examined for the effect of ERI on adverse health outcomes using the *PROCESS* macro for SPSS (38). When the bias correction *Bootstrap* sample size was set to 5,000 and the 95% confidence interval (CI) of the mediating effect did not contain 0, it was deemed statistically significant. A significance level of *α* = 0.05 (bilateral) was applied.

### 2.5 Ethical considerations

This study was approved by the Ethics Review Committee of West China Hospital of Sichuan University (Approval No.:2024.309). All participants were informed and voluntarily participated in the study.

## 3 Results

### 3.1 Characteristics of participants

A total of 1,540 emergency department nurses from 30 tertiary general hospitals in 20 provinces or autonomous regions were surveyed. The nurses had a mean age of 32.23 ± 6.80 years, with ages ranging from 20 to 58 years. Among the participants, 78.6% were female, predominantly married with children. Of the surveyed nurses, 403 (26.2%) reported experiencing ERI. The socio-demographic information, work characteristics, and lifestyle of the participants are detailed in Table 1.

**Table 1:** Characteristics of the participants (*n* = 1,540).

Variable	Category	*n*	%
Gender	Female	1,211	78.6
	Male	329	21.4
Marital status	Unmarried	560	36.4
	Married	980	63.6
Number of children	No	710	46.1
	Yes	830	53.9
Educational level	Associate degree	186	12.1
	Bachelor’s degree or above	1,354	87.9
Professional title	Junior RN	907	58.9
	Middle RN	574	37.3
	Senior RN	59	3.8
Years of nursing experience	1–2 years	242	15.7
	3–10 years	742	48.2
	11–20 years	421	27.3
	>20 years	135	8.8
Night shift	No	181	11.8
	Yes	1,359	88.2
Working hours per week	≤40 h per week	577	37.5
	41-48 h per week	780	50.6
	49-58 h per week	123	8.0
	≥59 h per week	60	3.9
Number of night shift	0	181	11.8
	1–4 times per month	239	15.5
	5–8 times per month	659	42.8
	>8 times per month	461	29.9
Smoking status	No	1,453	94.4
	Yes	87	5.6
Drinking status	No	1,355	88.0
	Yes	185	12.0

RN: registered nurse.

### 3.2 Correlation analysis of dimensions

The score for ERI among emergency nurses was 0.93 ± 0.57, the emotional exhaustion score was 11.30 ± 7.76, the WFC score was 42.48 ± 16.21, the score for somatic symptoms was 39.58 ± 13.61, and the sleep disorder score was 8.56 ± 3.12. ERI was correlated significantly positively with emotional exhaustion, WFC, somatic symptoms and sleep disorders (*r* = 0.624, 0.552, 0.554, 0.335). The scores are detailed in Table 2.

**Table 2:** Descriptive statistics and correlations for the main study variables.

Variable	Mean	SD	1	2	3	4	5	6	7
1. ERI	0.93	0.57	1						
2. Emotional exhaustion	11.30	7.76	0.624**	1					
3. Work–family conflict	42.48	16.21	0.552**	0.610**	1				
4. Somatic symptoms	39.58	13.61	0.554**	0.623**	0.655**	1			
5. Physical disorders	19.13	6.87	0.533**	0.575**	0.618**	0.975**	1		
6. Psychological disorders	20.46	7.08	0.547**	0.639**	0.660**	0.976**	0.903**	1	
7. Sleep disorders	8.56	3.12	0.335**	0.403**	0.435**	0.616**	0.572**	0.670**	1

**p < 0.01. SD: standard deviation; ERI: Effort-reward imbalance.

### 3.3 The mediating effect of emotional exhaustion on ERI and adverse health

The standardized data underwent mediation tests using Model4 in the *PROCESS* macro for SPSS. The results presented in Table 3 demonstrate that the positive predictive influence of ERI on somatic symptoms (*β* = 0.554, *t* = 26.080, *p* < 0.01), physical disorder (*β* = 0.534, *t* = 24.702, *p* < 0.01), psychological disorder (*β* = 0.547, *t* = 25.640, *p* < 0.01), and sleep disorders was statistically significant (*β* = 0.335, *t* = 13.961, *p* < 0.01). Moreover, following emotional exhaustion being utilized as a mediator variable in Table 4, the positive predictive influence of ERI on somatic symptoms (*β* = 0.271, *t* = 11.001, *p* < 0.01), physical disorder (*β* = 0.286, *t* = 11.111, *p* < 0.01), psychological disorder (*β* = 0.243, *t* = 9.985, *p* < 0.01), and sleep disorders remained significant (*β* = 0.137, *t* = 4.603, *p* < 0.01). And the emotional exhaustion also had positive predictive effect on somatic symptoms (*β* = 0.454, *t* = 18.467, *p* < 0.01), physical disorder (*β* = 0.397, *t* = 15.435, *p* < 0.01), psychological disorder (*β* = 0.488, *t* = 20.060, *p* < 0.01), and sleep disorders remained significant (*β* = 0.317, *t* = 10.700, *p* < 0.01) in Table 4. Furthermore, the results in Table 4 reveal that the 95% CIs of the *bootstrap* test for the direct effect of ERI on somatic symptoms (*β* = 0.271), physical disorder (*β* = 0.286), psychological disorder (*β* = 0.243), and sleep disorders (*β* = 0.137), as well as for the mediating effect of emotional exhaustion, did not encompass 0. This suggests that ERI may influence the negative health outcomes of emergency department nurses by affecting emotional exhaustion in Table 4.

**Table 3:** Regression analysis of the mediation model of emotional exhaustion.

Variable	Model	*β*	*t*	*R*	*R^2^*	*F*
Somatic symptoms	1	0.554	26.080**	0.554	0.307	680.188**
	2	0.624	31.309**	0.624	0.389	980.240**
	3	0.271	11.001**	0.658	0.433	585.794**
		0.454	18.467**			
Physical disorders	1	0.534	24.702**	0.533	0.284	610.203**
	2	0.624	31.309**	0.624	0.389	980.240**
	3	0.286	11.111**	0.617	0.38	471.273**
		0.397	15.435**			
Psychological disorders	1	0.547	25.640**	0.547	0.299	657.382**
	2	0.624	31.309**	0.624	0.389	980.240**
	3	0.243	9.985**	0.667	0.445	615.666**
		0.488	20.060**			
Sleep disorders	1	0.335	13.961**	0.335	0.113	194.898**
	2	0.624	31.309**	0.624	0.389	980.240**
	3	0.137	4.630**	0.417	0.174	161.888**
		0.317	10.700**			

**P < 0.01. Model 1: ERI predicting emotional exhaustion; Model 2: ERI predicting symptoms and sleep; Mondel 3: ERI and emotional exhaustion collaborative predicting symptoms, sleep. ERI: Effort-Reward Imbalance.

**Table 4:** The relationship between ERI, emotional exhaustion and health outcomes.

Model pathways	*B*	*SE*	95%*CI*		%
			LLCI	ULCI	
Somatic symptoms					
Total	0.554	0.021	0.512	0.595	
Direct	0.271	0.025	0.222	0.319	48.92
Indirect	0.283	0.019	0.248	0.321	51.08
Bootstrap					
ERI → Emotional exhaustion	0.624	0.043	0.553	0.723	
Emotional exhaustion →Somatic symptoms	0.454	0.028	0.398	0.507	
ERI → Somatic symptoms	0.271	0.031	0.209	0.333	
Physical disorders					
Total	0.534	0.022	0.491	0.575	
Direct	0.286	0.026	0.235	0.336	53.56
Indirect	0.248	0.018	0.213	0.285	46.44
Bootstrap					
ERI → Emotional exhaustion	0.624	0.042	0.558	0.723	
Emotional exhaustion → Physical disorders	0.397	0.028	0.343	0.452	
ERI → Physical disorders	0.286	0.030	0.226	0.346	
Psychological disorders					
Total	0.547	0.021	0.505	0.589	
Direct	0.243	0.024	0.195	0.291	44.42
Indirect	0.304	0.019	0.268	0.341	55.58
Bootstrap					
ERI → Emotional exhaustion	0.624	0.042	0.556	0.715	
Emotional exhaustion → Psychological disorders	0.489	0.028	0.431	0.542	
ERI → Psychological disorders	0.243	0.031	0.183	0.304	
Sleep disorders					
Total	0.335	0.024	0.288	0.383	
Direct	0.137	0.030	0.079	0.196	40.90
Indirect	0.198	0.020	0.159	0.238	59.10
Bootstrap					
ERI → Emotional exhaustion	0.624	0.042	0.554	0.719	
Emotional exhaustion → Sleep disorders	0.317	0.031	0.256	0.379	
ERI → Sleep disorders	0.137	0.032	0.079	0.203	

The table showed the total effect, direct effect, indirect effect, and Bootstrap path effect test. ERI: Effort-Reward Imbalance. SE: standard error; LLCI: lower limit confidence interval; ULCI: upper limit confidence interval.

### 3.4 WFC moderated mediation model effect test

**Figure 1:**
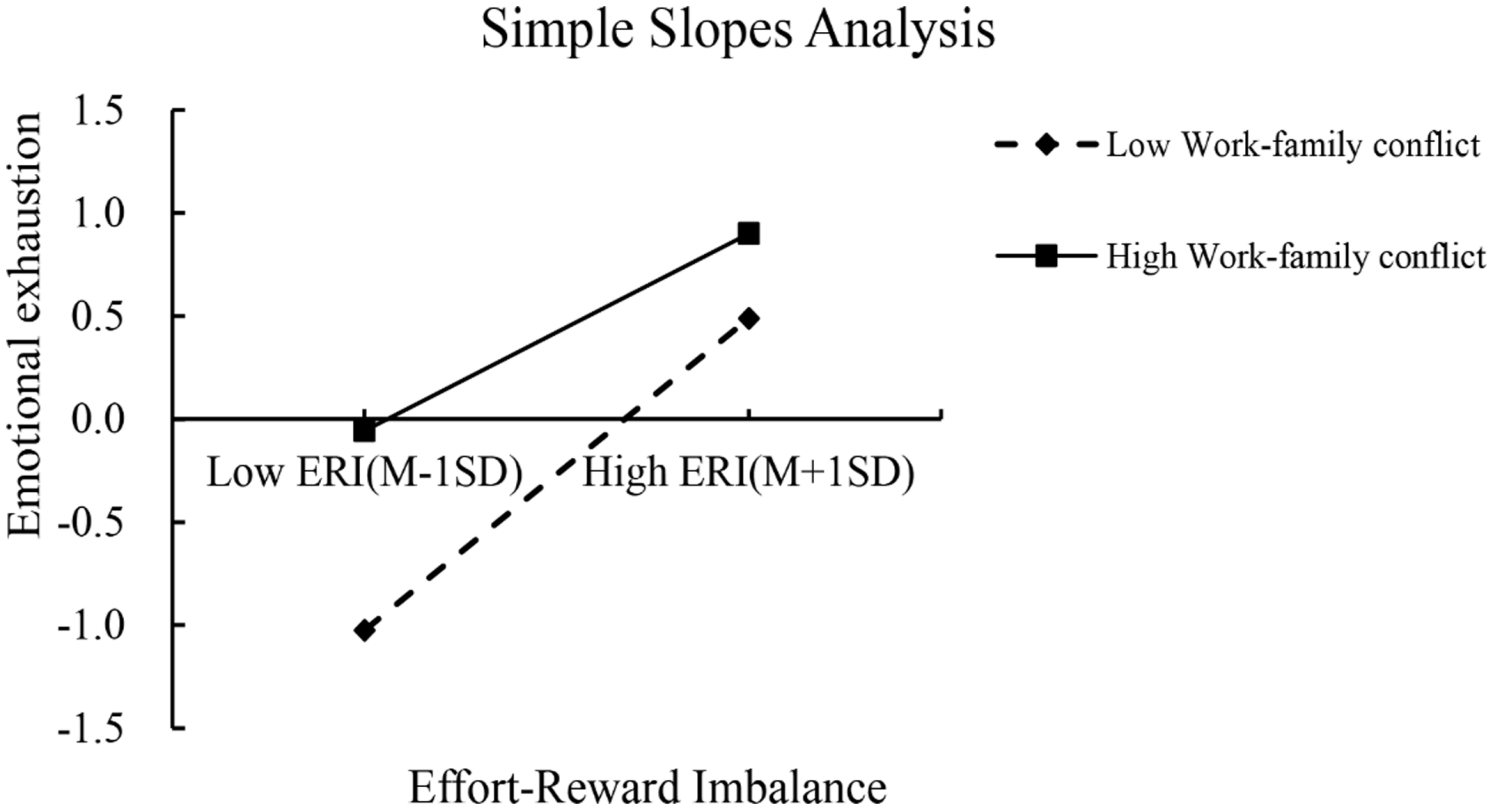
Simple slopes analysis of work–family conflict.

**Figure 2:**
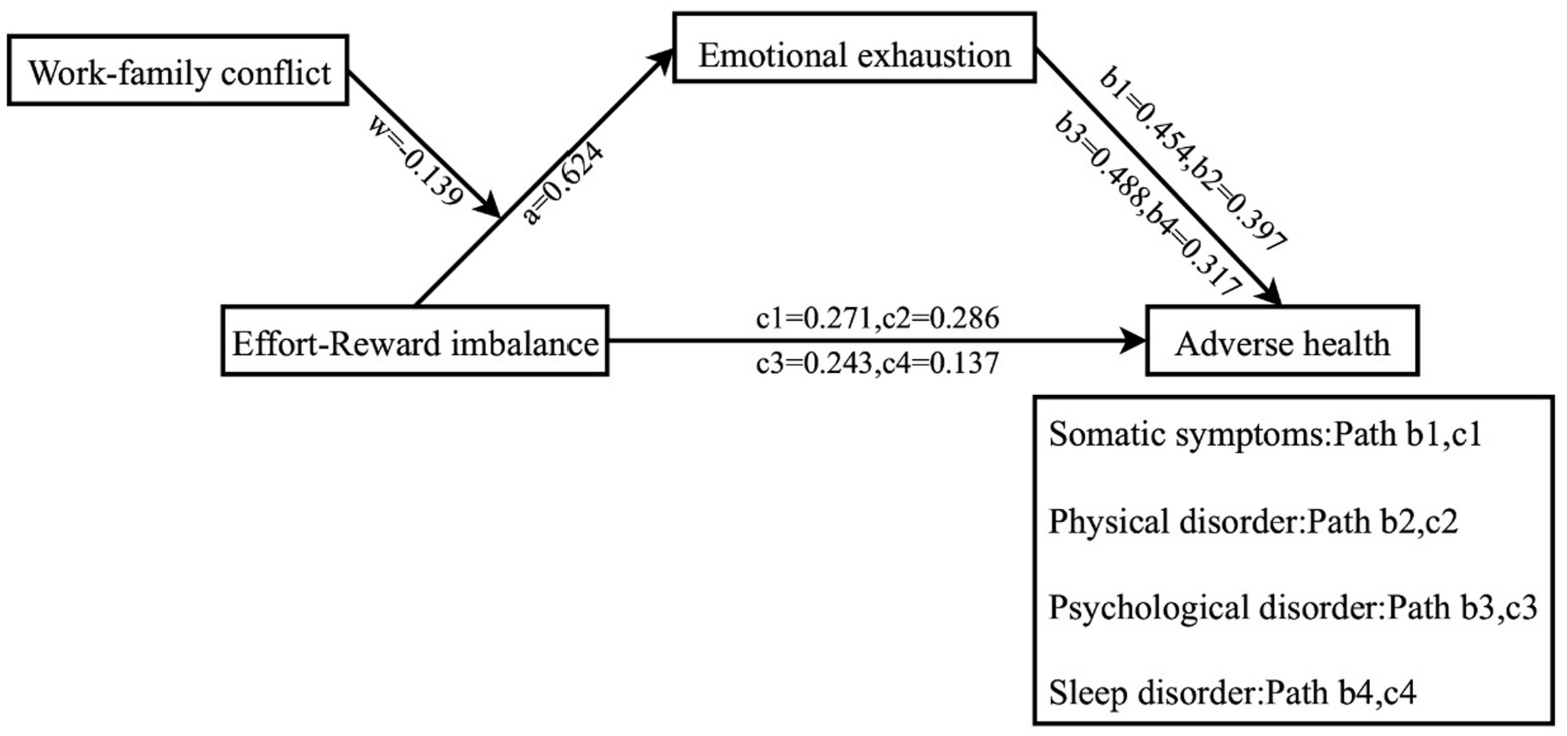
Moderated mediation models.

The mediated model with moderation was tested using Model7 in the *PROCESS* macro for SPSS assuming that the path X-M of the mediated model was moderated by WFC, in line with the theoretical model of the study. The results in Table 5 show that the interaction term between ERI and WFC significantly predicted emotional exhaustion (*β* = −0.139, *t* = −11.753, *p* < 0.01), indicating that work–family conflict moderates the influence of ERI on emotional exhaustion. Further simple slope analyses revealed that the relationship between ERI and emotional exhaustion was found to be significantly moderated by low levels of WFC (*simple slope* = 0.757, t = 21.035, *p* < 0.01) compared to high levels of WFC (*simple slope* = 0.479, *t* = 22.101, *p* < 0.01). Nurses with high levels of WFC showed a weaker moderated effect of ERI on emotional exhaustion than nurses with low levels of WFC. Additionally, the mediating effect of emotional exhaustion between ERI and somatic symptoms, physical disorder, psychological disorder, and sleep disorders also appeared to gradually decreases across three levels of WFC (*M-1SD*, *M*, and *M + 1SD*) in Table 6. This suggests that as WFC decreases among emergency department nurses, ERI is more likely to exacerbate emotional exhaustion, leading to somatic symptoms, physical disorder, psychological disorder, and sleep disorders in Figures 1, 2.

**Table 5:** Analysis of the moderating effect of work–family conflict.

Variable	*β*	SE	*t*	B	
				LLCI	ULCI
Constant	0.077	0.019	4.122**	0.040	0.113
ERI	0.618	0.027	22.681**	0.564	0.671
Work–family conflict	0.345	0.021	16.295**	0.303	0.386
ERI × Work–family conflict	−0.139	0.012	−11.753**	−0.162	−0.116
*R*	0.730				
*R^2^*	0.533				
*F*	584.124**				

**P<0.01; SE: standard error; LLCI: lower limit confidence interval; ULCI: upper limit confidence interval.

**Table 6:** Mediating effects at different levels of work–family conflict.

Variable	Effect	*β*	SE	95%*CI*	
				LLCI	ULCI
Somatic symptoms	Eff1(*M*-1SD)	0.344	0.028	0.292	0.402
	Eff2(*M*)	0.280	0.023	0.240	0.327
	Eff3(*M* + 1SD)	0.217	0.018	0.185	0.255
	Moderated mediation effects	−0.063	0.007	−0.077	−0.050
Physical disorders	Eff1(*M*-1SD)	0.300	0.027	0.250	0.356
	Eff2(*M*)	0.245	0.022	0.205	0.290
	Eff3(*M* + 1SD)	0.190	0.017	0.159	0.226
	Moderated mediation effects	−0.055	0.006	−0.068	−0.044
Psychological disorders	Eff1(*M*-1SD)	0.369	0.029	0.314	0.431
	Eff2(*M*)	0.301	0.024	0.258	0.350
	Eff3(*M* + 1SD)	0.233	0.019	0.199	0.272
	Moderated mediation effects	−0.068	0.007	−0.083	−0.053
Sleep disorders	Eff1(*M*-1SD)	0.240	0.027	0.190	0.296
	Eff2(*M*)	0.196	0.022	0.156	0.241
	Eff3(*M* + 1SD)	0.152	0.017	0.121	0.188
	Moderated mediation effects	−0.044	0.006	−0.056	−0.033

SE: standard error; LLCI: lower limit confidence interval; ULCI: upper limit confidence interval.

## 4 Discussion

The results revealed significant correlations between ERI, emotional exhaustion, and WFC with somatic symptoms and sleep disorders. Emotional exhaustion was found to mediate the link between ERI and somatic symptoms, as well as sleep disorders. Individuals should correctly view the relationship between effort and reward and avoid excessive investment. Meanwhile, organizations should increase the rewards given to employees in terms of salary, promotion prospects, and job security and reduce the workload.

In this study, the prevalence rate of ERI among emergency department nurses was 26.2%, which is lower than the overall prevalence rate of 68.7% among emergency department nurses reported in the most recent systematic review (39). Earlier studies indicated that the occurrence of ERI among general ward nurses in China ranged from 26.5 to 56.40% (8, 13). Previous studies have emphasized the prevalent challenges encountered by emergency department nurses, including unsustainable working conditions, shortages in human resources, and high workloads, which are consistent with the findings of Dong et al. (40). The demanding nature of their profession, especially during night shifts, leads to significant workloads for emergency department nurses. The necessity for 24/7 emergency medical care results in frequent shifts, which subsequently increases the occurrence of somatic symptoms and sleep disorders (41–43).

The study found a significant positive correlation between ERI by emergency department nurses and their somatic symptoms and sleep disorders. ERI is a key tool for assessing work-related stress, with research indicating that nurses facing high job stress have an 80% increased risk of major depressive episodes (44, 45). The study also revealed that ERI not only directly predicted anxiety symptoms but also correlated with burnout dimensions like emotional exhaustion and cynicism (46). This burnout, in turn, was linked to anxiety symptoms, highlighting a complex interplay between stress, burnout, and anxiety. Sleep disturbances among nurses in general hospitals were strongly linked to ERI, with sleep quality showing a negative correlation (47). Raju’s study further supported ERI as a predictor of poor sleep quality, underscoring the influence of occupational stress on nurses’ sleep health (48). The study’s findings stress the importance of physical and mental health and sleep quality interventions for emergency department nurses, given the strong connection between sleep disorders and work-related stress.

This study demonstrated that emotional exhaustion plays a mediating role between ERI and various health outcomes such as sleep disorders, somatic and psychiatric symptoms. ERI not only directly influenced these outcomes but also indirectly predicted them through emotional exhaustion, with a mediating effect of 59.10 and 51.08%, respectively. Consistent with previous research, emotional exhaustion was found to mediate the relationship between job stress and anxiety (49). Nurses, despite valuing their profession, reported stress as a significant factor contributing to feelings of frustration and exhaustion (50). As ERI increases, occupational stress rises, leading to burnout, depression, and lateral violence among nurses. Burnout, characterized by depersonalization, emotional exhaustion, and decreased personal fulfillment, is closely associated with perceived stress, particularly emotional exhaustion (51, 52). Previous studies have confirmed a strong correlation between ERI and emotional exhaustion (21, 53), a key component of burnout, within the nursing population. A systematic review of 67 articles on burnout and depression, as well as 34 articles on burnout and anxiety, demonstrated distinct and significant relationships between burnout and both anxiety and depression (54). Particularly, emotional depletion exhibited the highest effect sizes, reinforcing the mediating role of emotional depletion in the relationship between ERI and sleep disorders and somatic symptoms.

The moderated mediation analyses demonstrated that WFC moderated the strength of the relationship between ERI and somatic symptoms, sleep disorders mediated by emotional exhaustion. Nurses with high levels of somatic showed a weaker moderated effect of ERI on emotional exhaustion than nurses with low levels of somatic. More specifically, as somatic decreases among emergency department nurses, ERI is more likely to exacerbate emotional exhaustion, leading to somatic symptoms and sleep disorders, indicating a negative moderating influence. These results align with the study by Sugawara et al. on a cohort of Japanese nurses, where they observed that the relationships between emotional exhaustion, depression, and mental health became weaker with higher levels of somatic (55). According to Conservation of Resources theory, individuals aim to accumulate resources surplus as a precaution against potential future challenges. In reality, individuals juggle multiple roles within limitations of resource availability and distribution. To bolster their resource reserves, individuals focus on averting loss spirals and promoting gain spirals. Therefore, given the current medical and health conditions as well as working environment, nurses may prioritize career progression over family obligations to prevent depletion of valuable resources. Contrary to previous research, a survey of 964 nurses demonstrated a significant positive relationship between emotional exhaustion and WFC (56). Both work-to-family and family-to-work disruptions were positively correlated with emotional exhaustion (28). This indicates that women facing conflict between work and family are more likely to experience emotional exhaustion due to challenges in balancing work and family responsibilities (27). Longitudinal studies have consistently shown a significant positive influence of WFC on emotional exhaustion over time (57). Furthermore, high levels of WFC indirectly led to increased anxiety symptoms, with emotional exhaustion acting as a mediator (58). These findings suggest a link between WFC, emotional exhaustion, and anxiety symptoms. Galletta’s study with nurses supported these results by showing that emotional exhaustion levels rose with increased WFC (59). In contrast, Kida’s study revealed an intriguing trend: nurses with family roles experienced lower burnout levels compared to those without family responsibilities. This implies that family roles could serve as a protective factor against work-related emotional stress (60). The studies highlight the intricate relationship between WFC, emotional exhaustion, and occupational health. Discrepancies in findings may stem from variations in national contexts and study populations, underscoring the need for additional research in this area.

These results offered several important implications for nursing administration. Nursing managers should focus on improving the nursing work environment with good labor remuneration, and emotional support for emergency nurses (64). Developing a sound nursing career development system and providing a healthy work environment for emergency nurses. For example, nursing managers should proactively adjust the scheduling system for emergency nurses to reduce work stress and alleviate the work–family conflict (61, 62). These measures will not only help nurses maintain a healthy balance between their professional and personal lives but also improve the quality and safety of healthcare services, ultimately leading to better patient care.

### 4.1 Limitations

This study utilized a cross-sectional survey research design to conduct a one-time survey, rather than longitudinal sequential studies, which limited the ability to analyze how ERI influences nurse occupational health through emotional exhaustion over time. First, while the cross-sectional design provides valuable insights, it limits the ability to draw causal conclusions. Additionally, the reliance on self-reported data introduced potential bias. Although we fully respect the originality of the data, there might still be potential biases in statistical analysis. The focus on emergency nurses to investigate the mediation role of emotional exhaustion and the moderated effect of work–family conflict may not fully capture the variations in different clinical departments. Further validation of the research findings within nurse from different departments is recommended.

## 5 Conclusion

The findings of our study indicate that emotional exhaustion serves as a significant mediator in the relationship between ERI and negative health outcomes of emergency department nurses, while demonstrating a gradual decrease in the moderated influence of ERI on emotional exhaustion as WFC levels rose. Nursing administrators are encouraged to explore interventions such as individual counseling, reasonable shift system, adequate time off, and improving compensation for emergency department nurses to alleviate emotional exhaustion, ultimately enhancing their overall physical and mental health.

## Data Availability

The original contributions presented in the study are included in the article/supplementary material, further inquiries can be directed to the corresponding author.

## Footnotes

^1^ https://www.wjx.cn/
